# Binary Graft of Poly(acrylic acid) and Poly(vinyl pyrrolidone) onto PDMS Films for Load and Release of Ciprofloxacin

**DOI:** 10.3390/polym15020302

**Published:** 2023-01-06

**Authors:** Belén Santillán-González, Lorena Duarte-Peña, Emilio Bucio

**Affiliations:** 1 División de Ciencias Biológicas y de la Salud, Unidad Xochimilco, Universidad Autónoma Metropolitana, Calzada del Hueso 1100, Col. Villa Quietud, Delegación Coyoacán, Ciudad de México C.P. 04960 CDMX, Mexico; 2 Departamento de Química de Radiaciones y Radioquímica, Instituto de Ciencias Nucleares, Universidad Nacional Autónoma de México, Circuito Exterior, Ciudad Universitaria, Ciudad de México C.P. 04510 CDMX, Mexico

**Keywords:** polydimethylsiloxane, acrylic acid, vinyl pyrrolidone, ciprofloxacin, gamma radiation, drug-load and release

## Abstract

Polymers are versatile compounds which physical and chemical properties can be taken advantage of in wide applications. Particularly, in the biomedical field, polydimethylsiloxane (PDMS) is one of the most used for its high biocompatibility, easy manipulation, thermal, and chemical stability. Nonetheless, its hydrophobic nature makes it susceptible to bacterial pollution, which represents a disadvantage in this field. A potential solution to this is through the graft of stimuli-sensitive polymers that, besides providing hydrophilicity, allow the creation of a drug delivery system. In this research, PDMS was grafted with acrylic acid (AAc) and vinyl pyrrolidone (VP) in two steps using gamma radiation. The resulting material was analyzed by several characterization techniques such as infrared spectroscopy (FTIR), swelling, contact angle, critical pH, and thermogravimetric analysis (TGA), demonstrating the presence of both polymers onto PDMS films and showing hydrophilic and pH-response properties. Among the performed methods to graft, the loading and release of ciprofloxacin were successful in those samples obtained by direct irradiation method. Furthermore, the antimicrobial assays showed zones of inhibition for microorganisms such as *Staphylococcus aureus* and *Escherichia coli*.

## 1. Introduction

The development of new drug-modified delivery systems is booming in the pharmaceutical industry due to them offering greater advantages than conventional systems. For example, they can improve the solubility and absorption of active pharmaceutical ingredients (API) in the organism, and decrease the dosage, diminishing the side effects. Additionally, they can modify the API’s rate and site of action, and thus, the main objective is achieved. Thereby, it is important to create drug transporters, which help to achieve the objectives mentioned before. In this sense, polymers are proving to be one of the most desired materials for carrier design, especially in the field of nanotechnology as nanocarriers, because they can be used for the delivery of small therapeutic molecules to genes, and proteins, also in cancer therapy, diabetes treatment, anti-infections, etcetera, helping to improve their therapeutic efficiency. Generally, we can find a variety of carriers. Among the main polymeric nanocarriers, there are polymeric micelles, polymersomes, polymeric nanogels, polymeric nanocapsules, and dendrimers. In addition, there are lipid-based nanocarriers as nanoemulsions, phospholipid micelles, liposomes, solid lipid nanocarriers, and inorganic nanocarriers that include quantum dot, carbon nanotubules, gold nanoparticles, magnetic nanoparticles, and silicon nanoparticles. They all have different sizes, structures, and morphology and, therefore, different biomedical applications [1,2,3,4,5]. Polymers are macromolecules with a structure consisting of repeating units and their properties depend on the chemical structure (the nature of end groups, possible branches, and tacticity), chain length, molar weight distribution pattern, cohesive forces, density, crystallinity, molecular mobility, morphology, thermochemical and calorimetric properties, degradation, etcetera [6].

Polydimethylsiloxane (PDMS), also known as silicone, is a polymer, whose physical and chemical properties allow it to have widespread use in the biomedical industry. These properties include biocompatibility, thermal and chemical stability, chemically inert, gas permeability, good mechanical properties, simplicity to handle and manipulate, and thermal and electrical insulator. For all of the above, it’s a material that has been widely used in the development of micropumps, microvalves, catheter surfaces, dressings and bandages, optical systems, implants, microfluidic devices, devices for drug delivery, DNA sequencing, and clinical diagnostic, etcetera [7,8,9,10,11,12]. However, PDMS in biological environments can result in bacterial fouling or incompatibility due to its hydrophobic characteristics that help the adhesion of microorganisms [13,14]. The surface modification of this material is an alternative to improve their properties and create more complex systems with prophylactics characteristics that can take part in an active way in medical treatments. A promising alternative is the surface modification with materials that allow the controlled load and release of active agents through supramolecular interactions.

Poly acrylic acid (PAAc) is an anionic polymer, highly hydrophilic, sensitive to pH, and biocompatible [15], which has been used for the carrier systems fabrication of active agents as metallic nanoparticles or drugs [16]. On the other hand, polyvinyl pyrrolidone (PVP) is a highly biocompatible polymer used widely in the pharmaceutical industry for the development of various formulations, from tablets to nanofibers and contact lenses [17]. Additionally, due to its amphiphilic structure, it can interact with both hydrophilic and hydrophobic molecules; nonetheless, its application is limited for the low mechanical properties that show in its homopolymer form, so it tends to copolymerize or be used with other polymers, thus obtaining materials with better properties. The combination of PAAc and PVP has shown favorable results in the fabrication of hydrogels and the stabilization of drugs [18,19,20,21].

This work aims to propose the synthesis of a binary graft in two steps of acrylic acid (AAc) and 1-vinyl-2-pyrrolidone (VP) onto silicone films (PDMS) using gamma radiation as a physical initiator of the polymerization, to provide the surface hydrophilicity, biocompatibility and pH sensitivity, for its application in the load and release of ciprofloxacin. The ionizing radiation allows the formation of active dots along the polymer matrix, favoring the graft, and is an initiator that does not generate pollution in the final product, achieving high-purity materials [22].

## 2. Materials and Methods

### 2.1. Materials

PDMS films with a thickness of 1 mm were from Good-fellow (Huntingdon, UK). AAc, VP and ethanol were purchased from Aldrich Chemical, Saint Louis, MO, USA. The monomers AAc and VP were purified by vacuum distillation. Phosphate-buffered saline was prepared with salts from Aldrich Chemical, Saint Louis, MO, USA. For all assays, it was used distilled water. The gamma-ray source was a ^60^Co Gamma beam 651-PT (Nordion Ottawa, Ontario Canada Inc., Toronto, ON, Canada) proportioned by the Nuclear Science Institute at the National Autonomous University of México (ICN-UNAM).

### 2.2. Synthesis of PDMS-g-AAc

AAc grafted onto PDMS films was made by the direct irradiation method (DM), using gamma (γ) radiation. This method consists of the simultaneous irradiation of the polymeric matrix (PDMS) and the monomer to graft (AAc) under the atmosphere without oxygen.

PDMS films (6 × 1 cm) were weighed and placed in glass ampoules. AAc solutions at different concentrations were prepared: 20, 30, and 35% *v*/*v*, using ethanol: water (1:1) as solvent. To each ampoule 9 mL of AAc solution was added with the concentration chosen for each one and then they were bubbled with Ar for 20 min. Subsequently, the ampoules were sealed and subjected to γ radiation at doses of 20 and 30 kGy. After, the ampoules were opened, and the samples were removed to wash them with distilled water in order to remove the non-grafted homopolymer. Finally, the samples were dried in a vacuum oven, weighed, and the graft percentage was calculated according to Equation (1), where W_2_ is the grafted sample weight (g), and W_1_ is the initial weight of the sample (g).

(1) $$\mathrm{Graft}\;\left(\%\right)=\frac{\left(W_{2}{\;-\;W}_{1}\right)\;\times \;100}{W_{1}}$$

The graft procedure was taken from work carried out by Velazco-Medel, et.al., changing the parameters of monomer concentration and the applied dose [23]. 

### 2.3. Synthesis of (PDMS-g-AAc)-g-VP

VP grafted onto PDMS-*g*-AAc films was also made by employing the direct irradiation method with the methodology described in the previous Section 2.2. In this case, the factors to be modified were VP concentration [15, 20, 25, 30, 35, 40, 50% *v*/*v*] and the radiation dose applied (2.5, 5, 10, 15, 20, 25, 35 kGy). Distilled water was used as solvent.

Additionally, it was observed that PDMS-*g*-AAc showed interaction with VP, only using the factors of temperature and reaction time under and oxygen-free atmosphere. For which reason, tests without radiation (WR) were carried out, modifying the parameters: VP concentration [10, 20, 30% *v*/*v*], temperature (50, 60, 70 °C), and reaction time at 8, 16, and 24 h.

### 2.4. Infrared Spectroscopy (FTIR-ATR)

This technique was used to characterize the grafted samples and thus be able to determine the presence of the different functional groups. Analysis was performed using a PerkinElmer Spectrum 100 spectrometer (PerkinElmer Cetus Instruments, Norwalk, CT, USA) with 16 scans in the ATR module and in the 4000 to 650 cm^−1^ range.

### 2.5. Swelling

The dry samples were weighed and each one was placed in a glass with distilled water. A water bath at 25 °C was prepared, where the glasses were placed. Afterward, each certain time (5, 10, 15, 30 min., 1, 2, 4, 6, 8, 24, and 32 h), the samples were removed from the glasses and the excess of water was removed with blotting paper to be able to weigh them and return them to their corresponding glass. The procedure was repeated until it did not observe significant changes in the weight of the films. All the samples were measured in triplicate. Swelling percentage was determinate by Equation (2), where W_2_ is the swelled sample weight (g), and W_1_ is the initial weight of the sample (g).

(2) $$\mathrm{Swelling}\;\left(\%\right)=\frac{\left(W_{2}{-W}_{1}\right)\;\times \;100}{W_{1}}$$

### 2.6. Contact Angle

To confirm the hydrophilic or hydrophobic properties of grafts, their degree of wetting was measured by this technique. It was employed a goniometer DSA 100 Krüss GmbH, Hamburg, Germany. The angles of the drop onto the surface of the films were measured from time 0, 1, 5,10, 15, and 20 min until the moment which did not observe significant changes. All measurements were performed three times.

### 2.7. Critical pH

Phosphate buffer solutions were prepared at several pH (2.3, 3.3, 4.1, 5.5, 6.5, 8, and 10). The dry samples were weighed and each one was placed in a glass with a different pH solution for each day, starting with pH 2.3 and ending with pH 10. The glasses were placed in a water bath prepared at 25 °C. As in the swelling procedure, the samples were removed from the glasses and the excess of water was removed with blotting paper to be able to weigh them and return them to their corresponding glass. However, in this case, the procedure was repeated every 24 h (time limit of swelling found). All measurements were performed three times.

### 2.8. Thermogravimetric Analysis (TGA)

The analysis of thermal behavior of the PDMS and the grafted samples was monitored using a TGA Q50 (TA Instruments, New Castle, DE, USA), which works under a nitrogen atmosphere, the temperature range used was from 30 to 800 °C with a heating rate of 10 °C/min. 

### 2.9. Loading and Release of Ciprofloxacin for Spectrophotometer UV-Vis

#### 2.9.1. Ciprofloxacin Loading

The load of the drug was made for PDMS, [PDMS-g-AAc], [PDMS-g-AAc]-g-VP for DM, and [PDMS-g-AAc]-g-VP without radiation. The dry samples were weighed and placed in 3 mL of a solution of 12 µg/mL of ciprofloxacin in a water bath prepared at 25 °C. Monitoring of the loading was made with a spectrophotometer UV-Vis SPECORD 200 PLUS (Analytik Jena, Jena, Germany) at 266 nm, first at time 0 with the reference solution (distilled water) and then with the samples at time 2, 4, 6, 24, 30, 48, and 96 h. The quantification was performed with a calibration curve (Figure S1), and each measurement was performed in triplicate.

#### 2.9.2. Ciprofloxacin Release

The loaded samples were washed with distilled water and placed in 3 mL of a phosphate buffer dissolution (pH 7.4), in a water bath prepared at 37 °C and with oscillation at 130 rpm. The release monitoring was performed with the spectrophotometer UV-Vis, at 266 nm measuring at times 0, 0.5, 1, 2, 4, 6, 24, and 30 h. Carrying out a calibration curve to the quantification of the released drug (Figure S2). Furthermore, the statistic program Excel DDSolver software was used for the analysis of the release model.

### 2.10. Antimicrobials Assay: Agar Diffusion or Kirby-Bauer Method 

Samples of around 30 mg were inserted into a petri dish that contained Müeller-Hinton medium, sown with *Staphylococcus aureus* or *Escherichia coli*, at a concentration of 1.5 × 10^8^ CFU/mL and 24 h of grown. The samples were incubated for 24 h at 37 °C, and the zone of inhibition was measured from the center of the sample until the periphery where the bacterial grew was observed.

**Figure 1:**
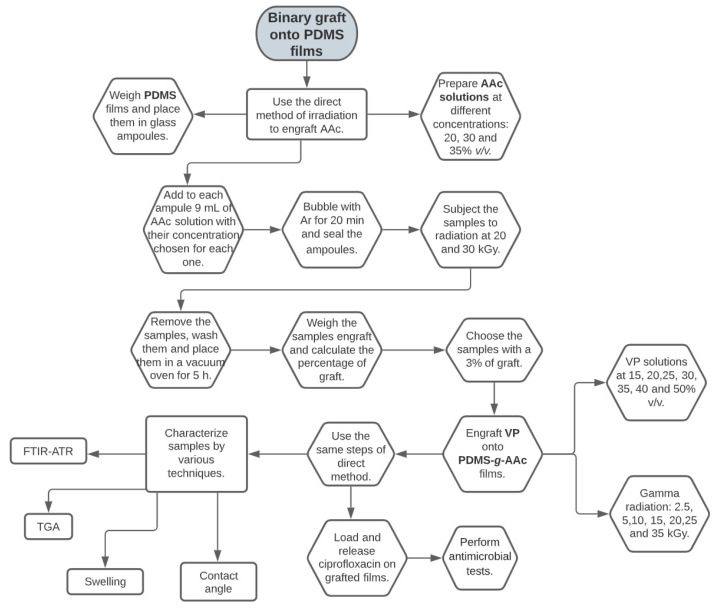
Experimental methodology.

Figure 1 shows a scheme of the experimental methodology developed during the work.

## 3. Results 

### 3.1. Synthesis of PDMS-g-AAc

**Figure 2:**
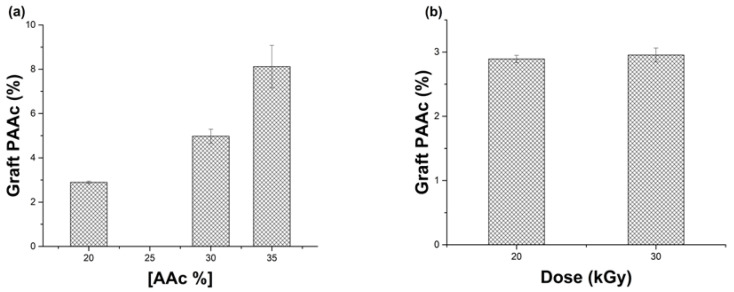
Percentages of PAAc graft onto PDMS films. (**a**) Effect of the monomer concentration and (**b**) Effect of the γ radiation doses. Reported: the mean ± standard error of the mean, *n* = 3.

In this synthesis, the factors that changed were the concentration of monomer to graft (AAc) at 20, 30, and 35% *v*/*v* and the dose of γ radiation at 20 and 30 kGy. Figure 2a shows that the higher concentrations increased the AAc graft. Nonetheless, the films modified with AAc concentrations of 35% presented a break when tried to manipulate. Concerning the dose, in Figure 2b, the graph exhibits that a significant difference regarding the percentage of graft for both doses (3%) did not exist, and this percentage allowed the manipulation of the films. With this in mind, it was decided to work with 20 kGy. The chosen final conditions were 20% *v*/*v* AAc concentration, Ar bubbling for 20 min, and a dose of 20 kGy to obtain materials with approximately 3% of the graft [PDMS-*g*-AAc (3%)].

### 3.2. Synthesis of [PDMS-g-AAc]-g-VP, Second Graft for the Direct Irradiation Method 

**Figure 3:**
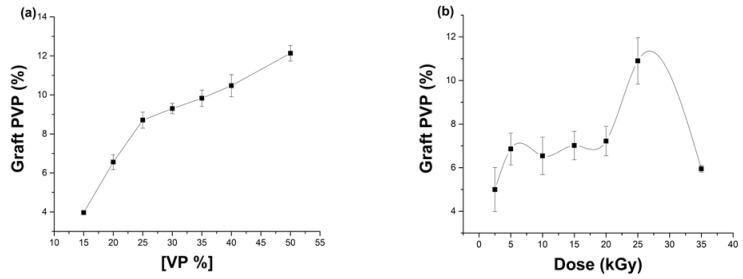
Synthesis of [PDMS-*g*-AAc]-*g*-VP by direct irradiation method (**a**) Effect of VP monomer concentration and (**b**) Effect of γ radiation doses. Reported: the mean ± standard error of the mean, *n* = 3.

In Figure 3a, it is noted that, as the concentration of VP increased, the higher the graft was. Concentrations of 30, 35, and 40 % showed graft values close to each other, 9.3 ± 0.3, 9.8 ± 0.4, and 10.5 ± 0.6%, respectively. On the other hand, the effect of the doses is shown in Figure 3b, the doses between 5 and 20 kGy did not present an apparent change as the graft remained at around 6 to 7%, but with a dose of 25 kGy there existed an increase until 11 ± 1.1% of graft; the opposite effect occurred when a dose of 35 kGy, where it was possible that there existed a matrix degradation, as the percentage decayed until 6%. The films tended to deform when the percentage of graft increases. Hence, it was decided to leave the conditions of VP to 30% and 10 kGy of dose, thereby this gives us results modifications around 10%.

### 3.3. Synthesis of [PDMS-g-AAc]-g-V, Second Graft for without Irradiation Method

**Figure 4:**
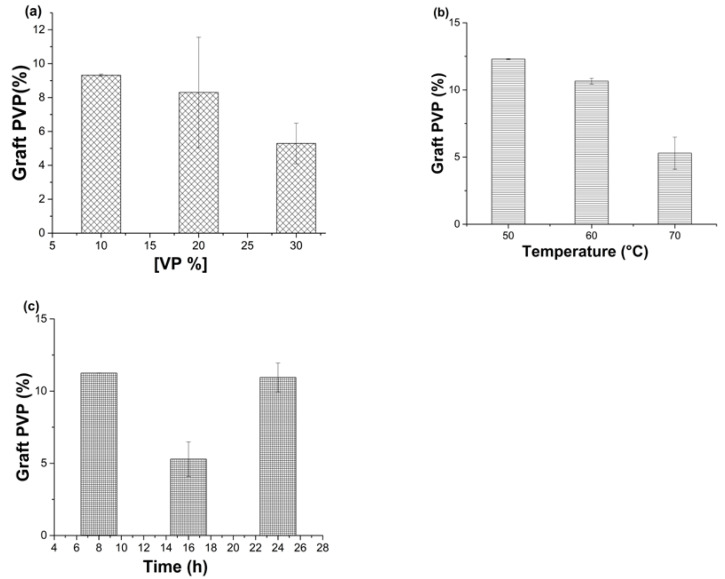
Percentages of graft of PVP onto films [PDMS-*g*-AAc], (**a**) Effect of VP concentration, (**b**) Effect of temperature, and (**c**) Effect of reaction time. Reported: the mean ± standard error of the mean, *n* = 3.

Figure 4a shows that lower concentrations of VP (10 and 20% *v*/*v*) produced higher graft percentages between 8 and 9%, while with 30% *v*/*v* VP concentration, the graft was minimum (5.3% ± 1.2). However, maintaining the same concentration (30% *v*/*v*) but diminishing the temperature at 50 °C, the graft increased to 12.3 ± 0.04% (Figure 4b). Finally, Figure 4c shows that there was no apparent difference in the percentage of VP grafted by varying the reaction time between 8, 16, and 24 h, so the key factor was the temperature. The conditions to obtain materials [PDMS-*g*-AAc (3%)]-*g*-VP (10%) were 30% *v*/*v* VP concentration, 50 °C, and 16 h. 

### 3.4. Infrared Spectroscopy (FTIR-ATR)

**Figure 5:**
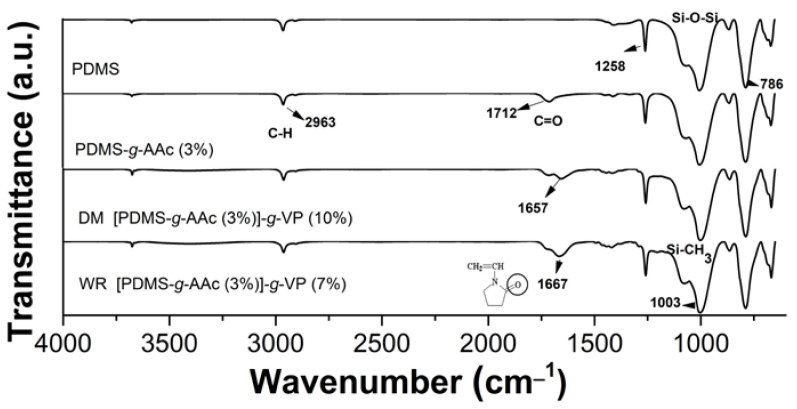
IR specters of samples PDMS without modification (up), first graft for DM (second from up to down), double graft for DM (third from up to down), and WR (down).

Figure 5 presents the infrared specters of different materials showing the functional groups expected for each sample. First, the polymeric matrix of PDMS showed bands between ~1000 y ~750 cm^−1^, corresponding to the Si–O–Si stretching and Si–CH_3_ vibrations. There was a short band attributed near 2900 cm^−1^, which is the typical band for C–H stretching [23]. In the [PDMS-*g*-AAc] spectrum, the band in 1712 cm^−1^ corresponded to the carbonyl group of AAc, confirming its presence on the film, although the bands of PDMS still appeared [24]. The next specters belonged to binary graft [PDMS-*g*-AAc]-*g*-VP, although each one was obtained for a different method, the band to ~1650 cm^−1^ that belonged to the carbonyl group of pyrrolidone appeared in both specters [25].

The IR technique proved the presence of both monomers onto PDMS, maintaining the functional groups of these in all of the specters and exhibited the characteristic functional group of AAc and VP.

### 3.5. Swelling

**Figure 6:**
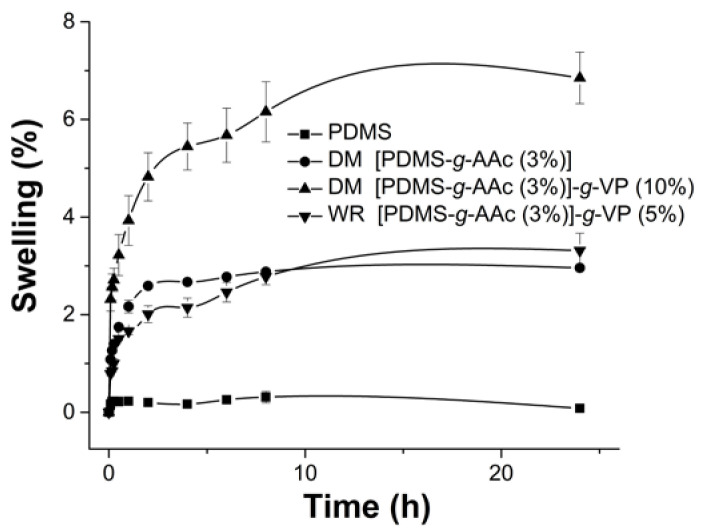
Swelling profiles of the samples PDMS, first graft with AAc and second graft obtained by DM and WR. Reported: the mean ± standard error of the mean, *n* = 3.

Figure 6 indicates that the PDMS did not present swelling, which coincides with its hydrophobic character; PDMS is an organic polymer, which is impermeable to water but soluble in nonpolar organic solvents [26]. On the other hand, the [PDMS-g-AAc] graft showed swelling (approximately 3%) due to the hydrophilic character given by the AAc, a characteristic given by its molecular structure that has a carboxyl group. The [PDMS-*g*-AAc]-*g*-VP films by both methods (DM and WR) had higher swelling due to the properties of the VP monomer, which is a polymer with hydrophilic and hydrophobic groups in its molecular structure that makes it soluble in various solvents [27]. The limit of swelling was found at 24 h, after which there were no significant changes in the weight of the films.

The fact that there is a double graft of AAc and VP on PDMS, increased its hydrophilic properties. Although the double grafts were obtained by both methods (DM and WR), it is observed that the swelling was more notorious in those samples obtained by DM. This fact shows that the employ of radiation allows a chemical copolymerization process to occur, whereas if not radiation is used, the result is a physical interaction.

### 3.6. Contact Angle

**Figure 7:**
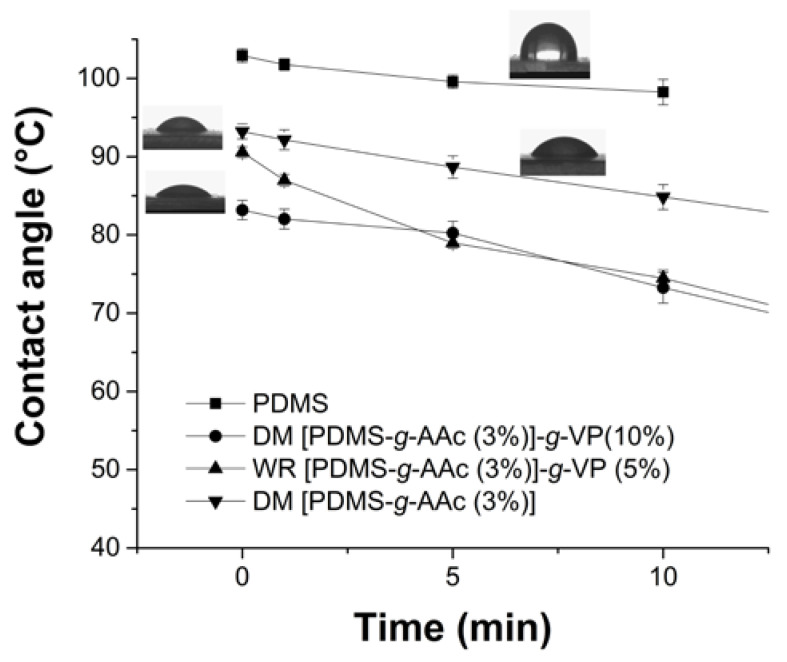
Contact angles of water for unmodified PDMS, PDMS-*g*-AAc (3%), and [PDMS-*g*-AAc (3%)]-*g*-VP (10%) with and without irradiation. Reported: the mean ± standard error of the mean, *n* = 3.

Figure 7 shows how to vary the water contact angles on the different materials. The PDMS changed from 103 ± 1° to 98 ± 2° in 10 min, so it did not change noticeably, confirming the hydrophobic property of this polymer [28]. In the sample with the first graft of AAc it was observed that the angle went from 93 ± 1° to 81 ± 3° in 15 min, which indicated that hydrophilicity was conferred to the PDMS film; however, the change was not high when compared to double grafts. The binary grafting by direct method showed a higher hydrophilic property than the other samples, the contact angle changed from 83 ± 1° to 67 ± 2°, due to the properties of both compounds on the base polymer giving it higher hydrophilicity. Something similar occurred for the double graft obtained without irradiation, but the hydrophilic property present in the film was slightly less, similar behavior to the swellings test, indicating a possible physical interaction. 

### 3.7. pH Sensitivity

**Figure 8:**
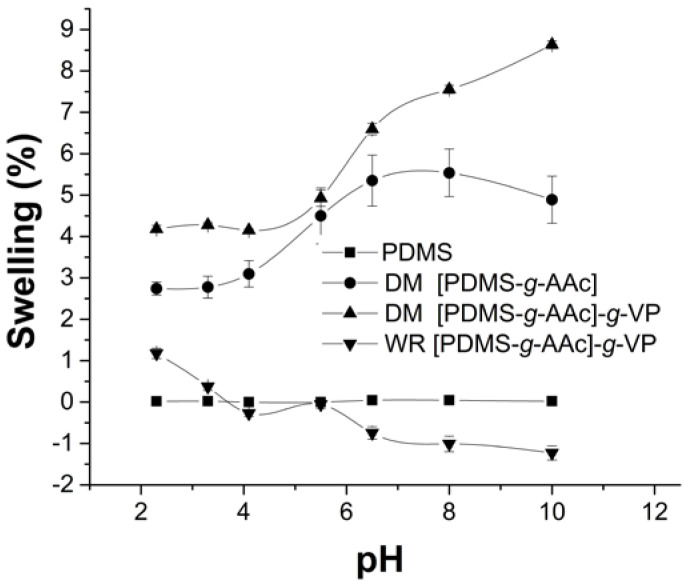
pH sensitivity tests after 24 h of swelling. Reported: the mean ± standard error of the mean, *n* = 3.

The pH sensitivity is a characteristic of ionic polymers, which, when there is a shift in pH in the environment, responds to this and causes change in the charge on the polymer chains, leading to swelling and drug release [29]. In this case, the PAAc is an anionic polymer with a pKa close to 4.3 [30]. Figure 8 shows that PDMS films did not have this behavior due to their hydrophobic properties. The PDMS grafted with AAc showed pH sensitivity with a critical pH of 5.3, meaning, the pH to which it responds, being the same as the material with binary graft [PDMS-g-AAc]-*g*-VP modified by direct irradiation method. However, the material modified without radiation did not show this characteristic, but a weight loss when changing the pH, which confirmed the physical nature of the interaction by this synthesis method. When changing the pH, the electrostatic balance of the material was altered, weakening the physical interaction and leading to weight loss.

### 3.8. Thermogravimetric Analysis

**Figure 9:**
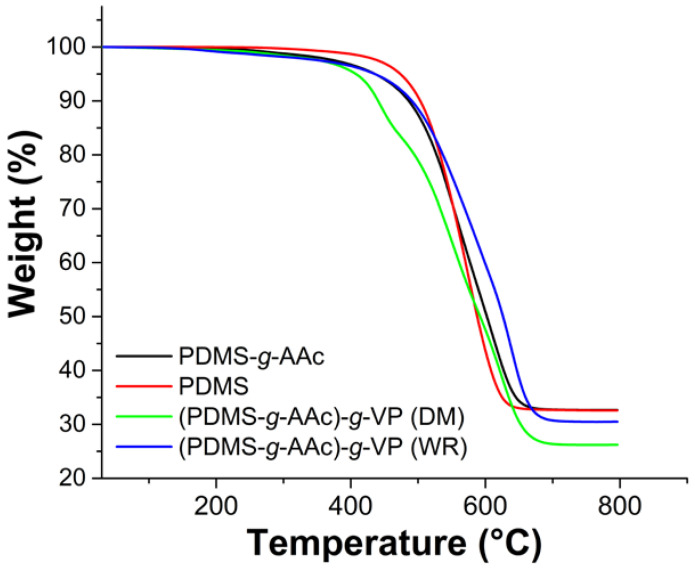
Thermograms of PDMS, PDMS-g-AAc (3%), and [PDMS-*g*-AAc (3%)]-*g*-VP (10%) with and without irradiation.

PDMS is a polymer with high thermal resistance whose temperature at 10% weight loss was approximately 503 °C, the material modified with AAc showed a decrease in this temperature to 486 °C, and the modification with the binary graft of AAc and VP caused a decrease in the stability with, at a temperature of 10%, weight loss of 441 °C. Finally, the (PDMS-*g*-AAc)-*g*-VP material modified without radiation did not show a significant difference at this temperature concerning the PDMS-*g*-AAc material, indicating that the interaction may have been physical. The decomposition temperature of PDMS was 574 °C; the material modified with AAc showed two decomposition temperatures at 550 °C for the decomposition of AAc and 640 °C for silicone. Finally, the modification with VP led to the apparition of an additional decomposition temperature at 442 °C (Figure 9) [31]. The derivative thermogravimetry curves are presented in Figure S3.

### 3.9. Loading and Release of Ciprofloxacin

**Figure 10:**
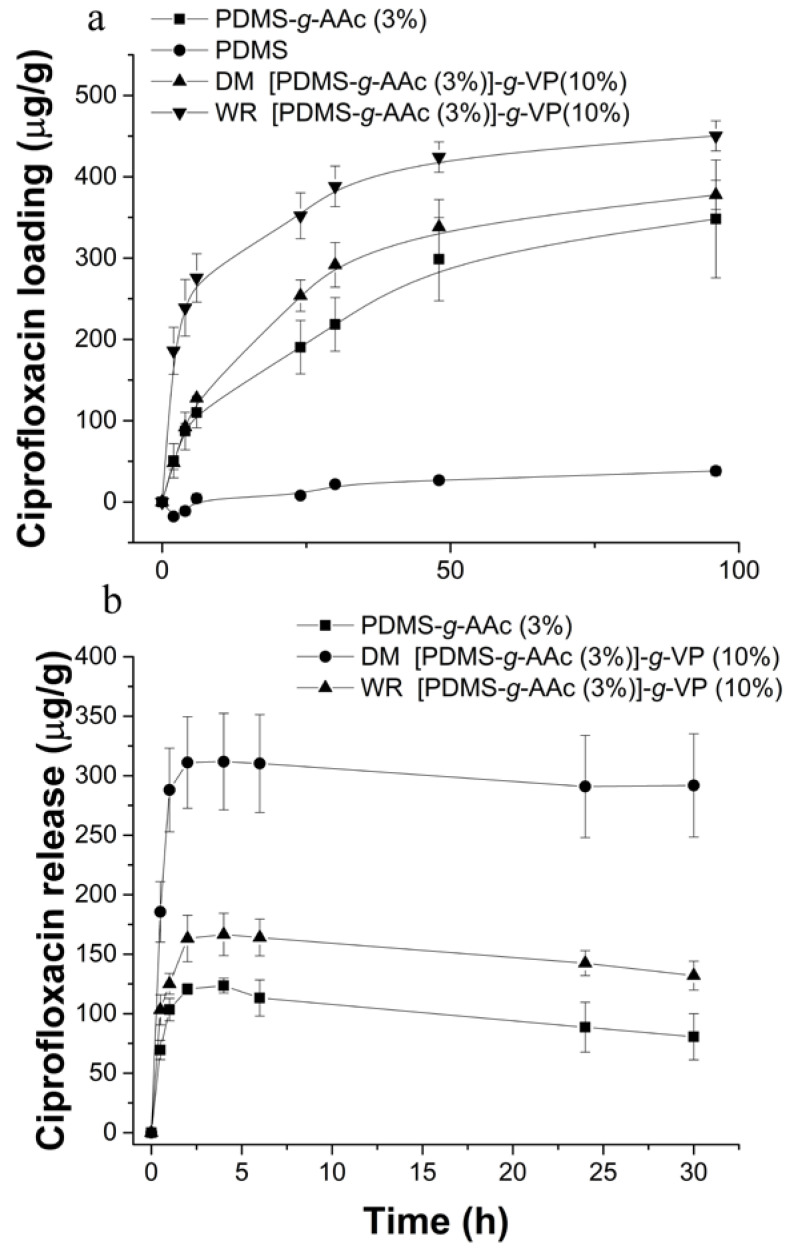
Drug delivery profiles, (**a**) the profiles of loading of ciprofloxacin, and (**b**) the profiles of the release of ciprofloxacin. By the UV-Vis spectrophotometry technique of the samples PDMS, PDMS-*g*-AAc, [PDMS-*g*-AAc]-*g*-VP by DM and WR. Reported: the mean ± standard error of the mean, *n* = 3.

Figure 10a shows the loading profiles for PDMS, PDMS-*g*-AAc (3%), and [PDMS-*g*-AAc (3%)]-*g*-VP (10%) with and without irradiation; it was observed that the PDMS during the 96 h loaded 38 ± 5.6 µg/g. When adding the first graft with AAc, the load increased to 348.14 ± 72.5 µg/g, and, finally, the double grafts had a higher drug load. However, the binary graft by the direct irradiation method reached a load of 378 ± 18 µg/g, and the one obtained by the method without irradiation had the highest load of 450 ± 18.5 µg/g. The behavior of the load profile indicated that, after 48 h, the load began to stabilize.

The release profiles of ciprofloxacin are shown in Figure 10b; the sample [PDMS-*g*-AAc (3%)]-*g*-VP (10%) synthesized by the direct irradiation method had a release percentage close to 100%, while the sample synthesized without irradiation released only 37%, a value near to the release obtained from PDMS-*g*-AAc, which was ~33%. All the materials presented maximum release at around 6 h. The PDMS without modification had no release.

From the data obtained for each sample, the release model that best fits each profile was searched using the DDSolver software. The three materials analyzed indicated, by their values of correlation index R^2^, Akaike information criterion (AIC), and the model selection criterion (MSC), that the best adjustment was to the Peppas-Sahlin model with ***T_lag_***, Equation (3). Where ***k*_1_** is related to the mechanism of diffusion, ***k*_2_** to the chain relaxation mechanisms, ***m*** is the Fick index, and ***T_lag_*** refers to the delay time [32,33].

(3) $$\frac{M_{t}}{M_{\infty }}=k_{1}{\left(t-T_{lag}\right)}^{m}+k_{2}{\left(t-T_{lag}\right)}^{2m}$$

Table 1 shows the parameters obtained for each sample. All the materials present values of ***k*_1_** greater than ***k*_2_**, indicating that the drug diffusion mechanism in the films was of the Fick type and not due to the relaxation of the polymer chains.

### 3.10. Agar Diffusion or Kirby-Bauer Method

**Figure 11:**
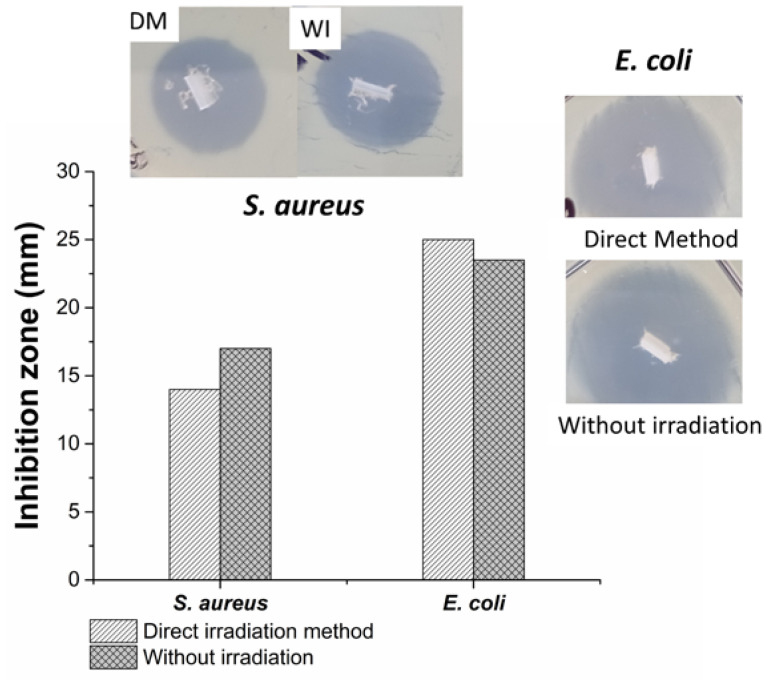
The zones of inhibition for [PDMS-*g*-AAc (3%)]-*g*-VP (10%) loaded with ciprofloxacin.

The PDMS samples, [PDMS-*g*-AAc (3%)] and [PDMS-*g*-AAc (3%)]-*g*-VP (10%), both by the direct method and without radiation, did not show a zone of inhibition. However, the binary grated materials loaded with ciprofloxacin showed inhibition zones for both *S. aureus* and *E. coli*. For gram-positive bacteria (*S. aureus*), the sample without irradiation presented the greatest inhibition zone of approximately 17 mm, while for gram-negative bacteria (*E. coli*), the sample with the binary graft by direct irradiation presented the largest zone of inhibition of about 25 mm (Figure 11). Comparing these results with the zones of inhibition of ciprofloxacin antibiograms loaded with 5 µg of drug, it was observed that the zones of inhibition coincided with bacteria not resistant to this drug that presented zones of inhibition >21 mm for *E. coli*, and for intermediate resistance in the case of *S. aureus* with a zone of inhibition between 16–20 mm [34,35]. This demonstrated that the material loaded with ciprofloxacin had antimicrobial activity. However, the amount of ciprofloxacin loaded and released was approximately 11 µg. Future studies should try to find an optimal load-release ratio for the material.

## 4. Discussion

Copolymerization is a process that improves the characteristics of a polymer. In this work, graft copolymerization with AAc and VP allowed us to provide PDMS with hydrophilic properties, giving it the ability to load and release ciprofloxacin, a broad-spectrum antimicrobial commonly used for the treatment of various infections, which gave it activity against *E. coli* and *S. aureus*.

**Figure 12:**
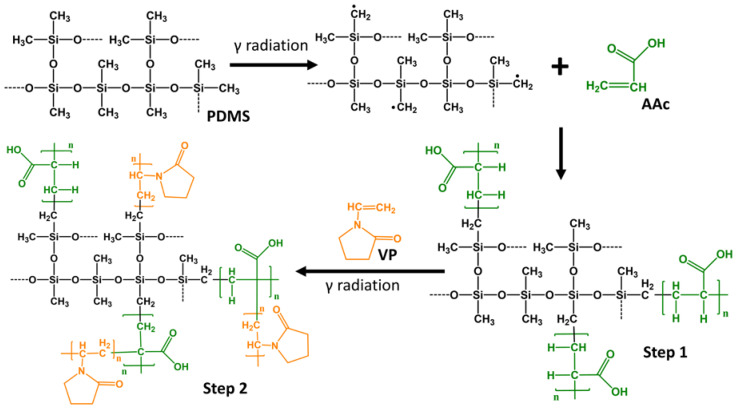
Schematic representation of the binary grafting formation. The black structure represents the PDMS, on the other hand, the green color refers to the AAc molecule and the orange color represents the VP molecule.

The direct radiation method proved effective for performing binary grafting in two steps, with graft percentages that allowed for manipulation and provided the ability to load drugs without deforming the films. Grafting of 3% AAc and 10% VP allowed the loading of ciprofloxacin and the subsequent release in a period of around six hours. When PDMS was exposed to gamma radiation, break of bonds occurred to forming radicals, which later allowed the grafting polymerization. The C–H bonds were more likely to break since they have the lowest binding energy, Figure 12 shows a schematic of the possible structure of this graft (step 1) [36]. For the second graft, not only the PDMS was irradiated but also the PAAc, which was why radicals were also created on the PAAc structure where the VP could also be grafted (Figure 12, step 2) [37].

On the other hand, the binary graft by the method without radiation was not very viable despite providing similar VP grafted percentages to the direct method. The pH sensibility test showed that the material did not have a completed chemical copolymerization because the material lost mass into the buffer solution. Additionally, in the UV-vis specter of the drug release test, a signal appeared before the label for the ciprofloxacin at 266 nm, possibly due to the loss of VP monomer or oligomer. Which demonstrated that the second graft with VP on PDMS-*g*-AAc films with only heating was not feasible since it may have been a physical and not a chemical interaction; VP will interact with acrylic acid forming hydrogen bonds that allow a highly stable physical interaction, which is affected by changing the electrostatic environment of the medium, for example, by changing the pH [38]. The swelling, contact angle, and pH-sensitive tests confirmed the hydrophilic property of the modified films, an important aspect for the purposes of this research, the loading and release of a drug that avoids bacterial contamination.

## 5. Conclusions

The binary grafting of AAc and VP on PDMS films was successfully performed using the direct irradiation method, obtaining percentages of 3% for the first graft and 10% for the second graft. The grafted material showed capability as a system for loading and releasing ciprofloxacin, which allowed it to have activity against *S. aureus* and *E. coli* in the agar diffusion assay with inhibition zones diameters of 14 and 25 mm for each bacterial strain, respectively. This system shows a potential application for the development of medical devices with antimicrobial characteristics; in addition, it gives the possibility of being a localized administration system since the drug is directly in the medical device.

## Figures and Tables

**Table 1:** Parameters of Peppas-Sahlin release model.

Sample	*k*_1_	*k*_2_	*M*	*T_lag_*
PDMS-*g*-AAc (3%)	44.7 ± 3.9	−14.4 ± 2.3	0.28 ± 0.02	0.29 ± 0.09
[PDMS-*g*-AAc (3%)]-*g*-VP (10%) DM	133.8 ± 14.3	−54.1 ± 4.6	0.14 ± 0.01	0.49 ± 0.01
[PDMS-*g*-AAc (3%)]-*g*-VP (10%) WR	39.5 ± 2.6	−10.8 ± 2.4	0.30 ± 0.07	xx

xx This material fits better for the traditional Peppas-Salhin model.

## Data Availability

Not applicable.

## Supplementary Materials

The following supporting information can be downloaded at: https://www.mdpi.com/article/10.3390/polym15020302/s1, Figure S1: Calibration curves to quantify load of ciprofloxacin. Figure S2: Calibration curves to quantify release of ciprofloxacin. Figure S3: DTG curve.
