# Supplementary material for: Binary Graft of Poly(acrylic acid) and Poly(vinyl pyrrolidone) onto PDMS Films for Load and Release of Ciprofloxacin
Source: Polymers (Basel). 2023 Jan 6;15(2):302. doi: 10.3390/polym15020302 (PMC9861813; doi:10.3390/polym15020302)
Supplement: Supplementary file 1 [file polymers-15-00302-s001.zip › polymers-2098447-supplementary.pdf]

Supplementary material

Binary Graft of Poly(acrylic acid) and Poly(vinyl pyrrolidone) onto PDMS Fims for Load and Release of Ciprofloxacin

Belén Santillán-González [1], Lorena Duarte-Peña [2,*], and Emilio Bucio [2,*]

[1] División de Ciencias Biológicas y de la Salud, Unidad Xochimilco, Universidad Autónoma Metropolitana, Calzada del Hueso 1100, Col. Villa Quietud, Delegación Coyoacán, Ciudad de México C.P. 04960 CDMX, Mexico

[2] Departamento de Química de Radiaciones y Radioquímica, Instituto de Ciencias Nucleares, Universidad Nacional Autónoma de México, Circuito Exterior, Ciudad Universitaria, Ciudad de México C.P. 04510 CDMX, Mexico

* Correspondence: lorena.duarte@correo.nucleares.unam.mx (L.D.-P.); ebucio@nucleares.unam.mx (E.B.)

1. Ciprofloxacin quantification and Derivative Thermogravimetry

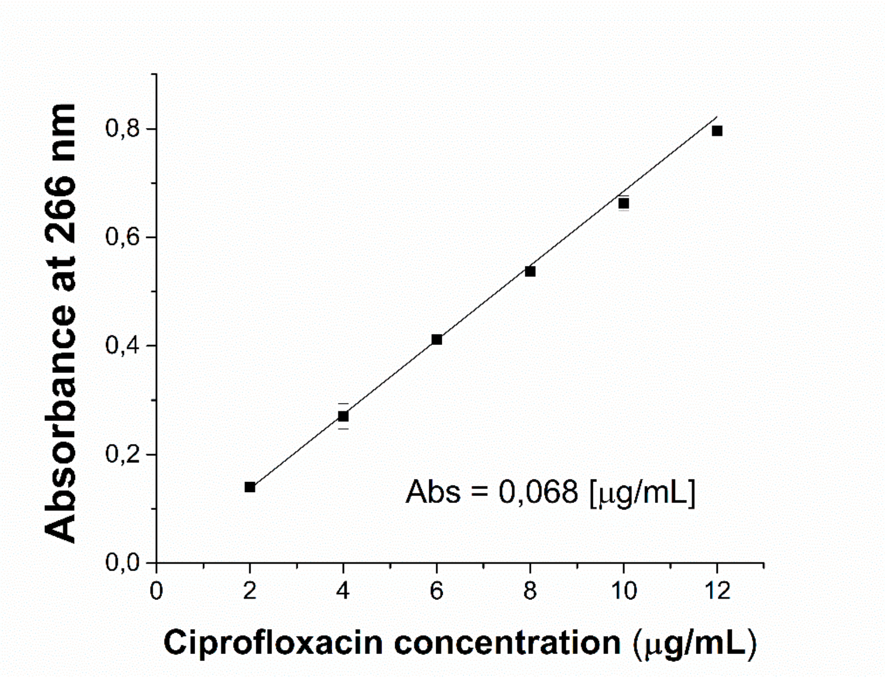

Figure S1. Calibration curves to quantify load of ciprofloxacin

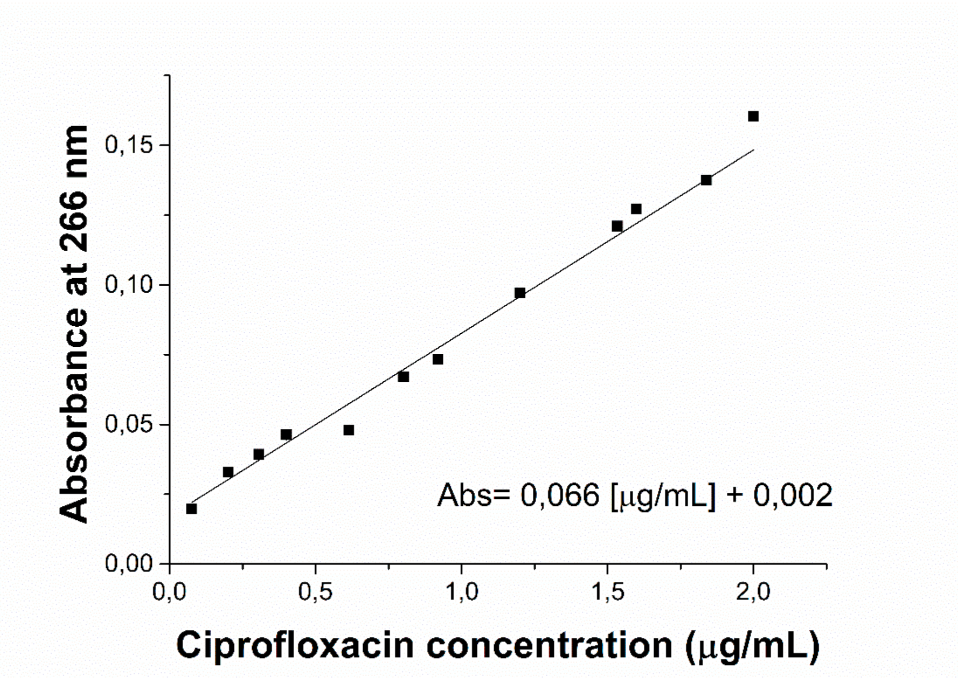

Figure S2. Calibration curves to quantify release of ciprofloxacin

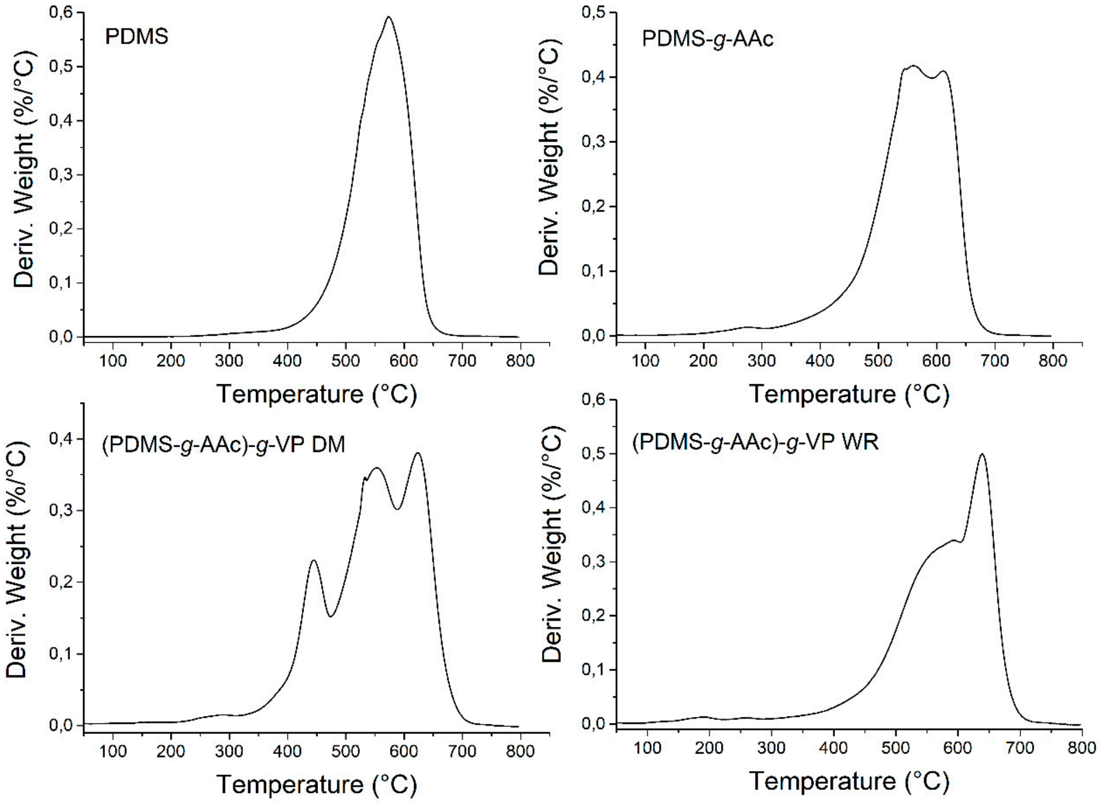

Figure S3. DTG curves
